# Integrating Soil Physicochemical Properties and Microbial Functional Prediction to Assess Land-Use Impacts in a Cold-Region Wetland Ecosystem

**DOI:** 10.3390/life15060972

**Published:** 2025-06-18

**Authors:** Junnan Ding, Shaopeng Yu

**Affiliations:** Heilongjiang Province Key Laboratory of Cold Region Wetland Ecology and Environment Research, Harbin University, Harbin 150086, China; wetlands1972@126.com

**Keywords:** land-use transition, soil microbial community structure, diversity, functional prediction, cold-region wetland, environmental drivers

## Abstract

This study investigated the effects of land-use change and wetland restoration on soil microbial community diversity, structure, and function in a cold-region wetland ecosystem. Soil samples from six land-use types were analyzed for key physicochemical and biochemical properties, including soil water content, pH, total nitrogen, soil organic carbon (SOC), and enzymatic activities. Significant differences in carbon and nitrogen availability were observed, with restored wetland soils showing higher SOC and moisture levels, while agricultural soils exhibited elevated nitrate concentrations. Bacterial community composition was estimated based on 16S ribosomal RNA gene sequencing, and microbial functional profiles were predicted using Functional Annotation of Prokaryotic Taxa (FAPROTAX) and BugBase. Bacterial communities were dominated by *Proteobacteria*, *Actinobacteriota*, and *Acidobacteriota*, with significant shifts among land-use types. Redundancy analysis revealed that SOC, SWC, total nitrogen (TN), and pH were key drivers of community differentiation. Functional prediction showed enrichment of fermentation and anaerobic metabolism in restored wetlands, while aerobic carbon metabolism dominated in agricultural and forest soils. These findings demonstrate that wetland restoration improves both taxonomic and functional diversity. While ecosystem multifunctionality and resilience were not directly quantified, the observed increases in microbial richness, functional group diversity, and enzymatic activity suggest enhanced ecological capacity and potential for system stability in cold-region wetlands.

## 1. Introduction

Cold-region wetland ecosystems, primarily distributed in boreal and cold-temperate zones, play vital roles in global carbon storage, hydrological regulation, and biodiversity maintenance [1]. These regions are characterized by long, cold winters, short growing seasons, and soils subject to seasonal freezing, making them especially sensitive to external disturbance. Globally, over 35% of wetlands have been lost since 1970, and China has experienced an estimated 23% decline in natural wetland area in recent decades [2]. This decline is largely driven by land-use change, namely, the conversion of natural wetlands into agricultural land, forestry plantations, or urban infrastructure, which often results in habitat fragmentation, soil degradation, and altered ecosystem processes [3,4]. The Sanjiang Plain of northeastern China exemplifies this trend. Historically dominated by expansive marshes, the region has undergone widespread drainage and reclamation over the past 50 years, becoming one of China’s most productive agricultural areas at the expense of severe wetland degradation [5]. Climate change compounds these effects. Rising temperatures and altered precipitation patterns affect wetland hydrology and accelerate biogeochemical transformations [6,7,8]. Experimental warming studies have shown that even moderate increases in temperature alter soil microbial activity, nutrient turnover, and carbon dynamics in boreal and temperate wetland soils [9].

Degradation of wetlands whether through drainage, cultivation, or climate-induced drying alters vegetation composition, oxygen availability, and soil moisture. These changes accelerate organic matter decomposition, reduce soil carbon and nitrogen pools, and shift nutrient cycling pathways [10,11,12,13,14]. In cultivated wetland soils, moisture is reduced while nutrient inputs increase due to fertilization, leading to a distinct soil environment from natural, peat-accumulating wetlands [15,16]. These physicochemical transitions directly influence soil microbial communities, which are critical regulators of ecosystem function [17,18]. Microbial diversity and community composition are highly responsive to changes in soil moisture, pH, nutrient availability, and vegetation inputs [19,20,21,22]. For example, land-use change from marsh to cropland often favors aerobic taxa such as Actinobacteria, while waterlogged conditions promote anaerobic microbes that drive methanogenesis and denitrification [23,24,25]. In cold-region wetlands, climate warming adds further stress, reorganizing microbial networks and potentially enhancing or disrupting nutrient processing [26,27,28]. Recent studies suggest that although hydrological and vegetative conditions may recover through restoration, microbial communities often show delayed structural and functional recovery due to legacy effects on soil structure, nutrient status, and redox potential [29,30,31]. For instance, a 10-year restoration effort in northeastern China restored vegetation and water regimes, yet microbial functional profiles remained distinct from natural wetlands [32]. These findings highlight that microbial recovery may lag behind other indicators of wetland restoration, warranting greater attention in restoration monitoring.

Despite growing interest in microbial responses to land-use and climate change, most studies have focused on isolated components such as carbon sequestration, vegetation succession, or single land-use transitions. Few have integrated soil physicochemical data, microbial taxonomic structure, and predicted microbial functions across full degradation-to-restoration gradients in cold-temperate regions [33,34,35]. To address this gap, a holistic approach is needed that captures how soil microbial communities respond to disturbance and recovery along land-use gradients and retrogressive succession; that is, the transition from intensively managed land back toward natural wetland conditions [36]. With advances in 16S rRNA gene sequencing, tools like FAPROTAX and BugBase allow for trait-based functional inference of microbial communities without requiring full metagenomic sequencing, offering insight into functional traits such as methane metabolism, nitrogen cycling, and stress tolerance [37].

This study was conducted in the Raolihe National Nature Reserve, a representative cold-temperate wetland ecosystem in northeastern China. The reserve encompasses a mosaic of land-use types resulting from both historical wetland loss and recent restoration. We selected six distinct land-use categories: natural forest (NF), plantation forest (PF), soybean farmland (SF), maize farmland (MF), soybean-retired meadow wetland (SRM), and maize-retired marsh wetland (MRM). These categories represent a gradient of anthropogenic disturbance and ecological recovery commonly observed in cold-region wetlands globally. This integrated approach enables us to evaluate how microbial taxonomic and functional structures respond to land-use change and how these changes may influence ecosystem resilience. Overall, our goal is to provide insights into the soil–microbe–environment interactions that govern wetland degradation and restoration in cold-region ecosystems. To address the above knowledge gaps, this study was conducted in the Raolihe National Nature Reserve, a representative cold-temperate wetland ecosystem in northeastern China, and was designed with three primary objectives and corresponding hypotheses. (1) The soil physicochemical properties and microbial community structures were compared across six distinct land-use types, including natural wetlands, agricultural lands, and restored sites. It was hypothesized that land-use intensification reduces soil nutrient availability and microbial diversity, whereas wetland restoration partially restores soil quality and microbial structural complexity. (2) Microbial diversity patterns (alpha and beta diversity) and predicted functional composition were assessed along a retrogressive wetland succession gradient, representing a shift from intensively managed cropland toward natural wetland conditions. It was hypothesized that microbial diversity and functional redundancy increase progressively along the restoration trajectory, indicating ecological recovery of microbial communities. (3) The relationships between environmental factors (soil physicochemical and biochemical attributes) and microbial community structure and function were evaluated. It was hypothesized that microbial community assembly is primarily driven by soil carbon availability and nitrogen forms, which regulate microbial niche differentiation and functional potential across land-use gradients. Achieving these objectives will improve our understanding of how land-use changes impact wetland soil ecology and will provide scientific guidance for wetland conservation and sustainable land management in cold-region ecosystems.

## 2. Materials and Methods

### 2.1. Site Description

This study was conducted in the Raolihe National Nature Reserve, located in northeastern Heilongjiang Province, China (48°45′–49°00′ N, 133°30′–133°50′ E), within the cold-temperate zone of the Sanjiang Plain. The region features a continental monsoon climate characterized by long, cold winters, short growing seasons, and seasonally frozen soils. The average annual temperature is approximately 1 °C, with a frost-free period of ~90 days and mean annual precipitation of 600 mm, mainly concentrated in summer. Dominant soils include meadow, gley, and peat soils, typical of lowland wetlands. Since 2014, a large-scale cropland-to-wetland restoration program has converted over 3000 hectares of farmland back to marshes through rewetting and ecological engineering. These efforts have resulted in a diverse mosaic of land-use types that reflect varying degrees of anthropogenic disturbance and different stages of ecological recovery in cold-region wetlands. To capture the ecological variability and assess the impact of land-use transformation on soil physicochemical properties and microbial functional profiles, six representative land-use types were selected. These include: (1) Natural Forest (NF), representing undisturbed conditions, characterized by mixed coniferous broadleaf stands dominated by *Larix gmelinii*, *Betula platyphylla*, and *Populus davidiana*, with understory species such as *Viburnum burejaeticum* and *Maianthemum bifolium*; (2) Plantation Forest (PF), artificial larch (*Larix gmelinii*) forests established on former wetlands, indicative of semi-natural managed systems; (3) Soybean Farmland (SF), long-term cultivated agricultural land planted with *Glycine max* L., subject to regular tillage and fertilization; (4) Maize Farmland (MF), intensively managed land dominated by *Zea mays* L.; (5) Soybean-Retired Meadow Wetland (SRM), previously cultivated soybean fields that have undergone passive wetland restoration through hydrological re-regulation, now dominated by meadow vegetation such as *Carex appendiculata*, *Calamagrostis angustifolia*, and *Glyceria spiculosa*; (6) Maize-Retired Marsh Wetland (MRM), restored from maize fields into saturated marsh environments via ecological engineering, characterized by obligate wetland plants including *Phragmites australis*, *Typha angustifolia*, and *Scirpus triqueter*. This land-use gradient from forest to agriculture to restored wetlands provides a comprehensive framework to evaluate microbial and soil ecosystem responses to disturbance and recovery [38,39]. To ensure biological replication, three independent sampling plots (each at least 100 m apart) were established for each land-use type. In each plot, twelve soil cores were collected and composited to form one sample, resulting in three composite biological replicates per land-use type (*n* = 3). This design allowed for true replication at the treatment level. All statistical analyses were performed using these biological replicates, and differences among land-use types were assessed using one-way ANOVA followed by multiple comparison procedures where applicable.

### 2.2. Sample Collection

In September 2022, soil sampling was conducted for six representative land-use types within the Raolihe National Nature Reserve. For each type, three independent 20 m × 20 m plots were established as biological replicates (n = 3 per type). Within each plot, twelve spatially distributed subsamples were collected from the 0–20 cm topsoil layer following an S-shaped (semi-randomized) sampling pattern designed to reduce spatial bias and ensure site coverage. Surface litter, roots, and stones were removed in the field. The twelve subsamples per plot were homogenized and pooled into a single composite sample, resulting in a total of 18 composite soil samples for downstream analysis. This design allows statistically robust comparisons across land-use types. All samples were sealed on site and transported to the laboratory under refrigerated conditions (~4 °C). In the laboratory, each composite sample was divided into two portions: approximately 10 g of fresh soil was placed into a sterile 50 mL centrifuge tube and stored at −80 °C for microbial analysis, while the remaining portion was air-dried, sieved through a 2 mm mesh, and homogenized for soil physicochemical analysis. Both microbial and chemical analyses were performed in technical triplicates to ensure measurement accuracy and reproducibility.

### 2.3. Analysis of Soil Physicochemical Properties

Soil physicochemical properties were determined following standardized laboratory procedures. Soil pH was measured on air-dried, 2 mm-sieved soil in a 1:2.5 soil-to-water suspension using a digital pH meter (SevenCompact S220, Mettler Toledo, Greifensee, Switzerland), calibrated with buffer solutions at pH 4.0, 7.0, and 10.0. Soil water content (SWC, %) was determined gravimetrically by drying fresh soil at 105 °C to a constant weight in a forced-air oven (DHG-9140A, Shanghai Yiheng Scientific Instrument Co., Shanghai, China). Soil organic carbon (SOC, g/kg) was analyzed using the Walkley–Black wet oxidation method with potassium dichromate and sulfuric acid [40]. Total nitrogen (TN, g/kg) was measured using the Kjeldahl digestion method with concentrated H_2_SO_4_ and a digestion block (FOSS Tecator Digestion System 2020, FOSS, Höganäs, Sweden) [41]. Dissolved organic carbon (DOC) was extracted from fresh soil using 0.5 M K_2_SO_4_ (soil/solution = 1:5, *w*/*v*), filtered through 0.45 μm membrane filters (Millex, Millipore, Burlington, MA, USA), and quantified using a total organic carbon analyzer (TOC-L CPH, Shimadzu, Kyoto, Japan) via high-temperature catalytic combustion [42]. Microbial biomass carbon (MBC) and microbial biomass nitrogen (MBN) were determined using the chloroform fumigation-extraction method. Fresh soil samples were fumigated with ethanol-free chloroform in a vacuum desiccator for 24 h, then extracted with 0.5 M K_2_SO_4_ and filtered for analysis. Extractable C and N were measured using the TOC analyzer and a total nitrogen module (TNM-L, Shimadzu, Kyoto, Japan), and MBC/MBN values were calculated using appropriate kEC and kEN conversion coefficients [43]. Ammonium nitrogen (NH_4_^+^-N) and nitrate nitrogen (NO_3_^−^-N) were extracted using 2 M KCl solution (soil:solution = 1:5), filtered, and analyzed by continuous flow injection analysis (AA3, SEAL Analytical, Norderstedt, Germany) [44]. Soil enzyme activities were determined to assess microbial functional potential. Urease activity was measured by incubating soil samples with urea substrate solution at 37 °C for 2 h, followed by quantification of released NH_4_^+^ using colorimetry (UV-6100, MAPADA, Shanghai, China) [45]. β-Glucosidase activity was measured using p-nitrophenyl-β-D-glucopyranoside (pNPG) as substrate, with p-nitrophenol release quantified at 400 nm [46]. Cellulase activity was determined using carboxymethyl cellulose (CMC) as substrate, and the amount of reducing sugars was measured using the dinitrosalicylic acid (DNS) method [47].

### 2.4. DNA Extraction and High-Throughput 16S rRNA Gene Paired-End Sequencing

Genomic DNA was extracted from 0.5 g of soil using the Omega E.Z.N.A.^®^ Soil DNA Kit (Omega Bio-Tek, Norcross, GA, USA) following the manufacturer’s protocol. The quality and purity of the extracted DNA were assessed using a NanoDrop 2000 spectrophotometer (Thermo Fisher Scientific, Waltham, MA, USA), measuring A260/A280 ratios to ensure DNA purity. Additionally, agarose gel electrophoresis (1% *w/v*) was performed to check the DNA integrity. A two-step polymerase chain reaction (PCR) was carried out on a GeneAmp 9700 PCR system (Applied Biosystems, Foster City, CA, USA). In the first step, universal primers 515F (5′-GTGCCAGCMGCCGCGGTAA-3′) and 907R (5′-CCGTCAATTCMTTTRAGTTT-3′) were used to amplify the V3–V4 region of the bacterial 16S rRNA gene. In the second step, barcodes were added. The PCR mixture (25 μL) contained 1× PCR buffer, 1.5 mM MgCl_2_, 0.2 mM dNTPs, 0.5 μM of each primer, 1.25 U of Taq DNA polymerase (Takara, Kusatsu, Japan), and 1 μL of template DNA. PCR conditions included an initial denaturation at 95 °C for 3 min, followed by 25 cycles of denaturation at 95 °C for 30 s, annealing at 55 °C for 30 s, and extension at 72 °C for 45 s, with a final extension at 72 °C for 10 min. To assess potential PCR inhibitors, a dilution series was performed for a subset of DNA extracts, ensuring that PCR amplification efficiency was not compromised. The PCR product was purified using a PCR cleanup kit (Omega Bio-Tek, Norcross, GA, USA) and quantified with a QuantiFluor^®^-ST fluorometer (Promega, Madison, WI, USA) before being adjusted for sequencing. The samples were then sent to Shanghai Meiji Biotechnology Co., Ltd. (Shanghai, China) for sequencing on the Illumina HiSeq 2500 PE250 platform (San Diego, CA, USA) for high-throughput sequencing [48]. All the sequences were deposited in the NCBI, and the serial number was PRJNA1271745.

### 2.5. Sequencing Data Processing and Analysis

Sequencing data were processed using QIIME 2 (version 2023.2) (Caporaso Lab, Northern Arizona University, Flagstaff, AZ, USA) in combination with the DADA2 plugin (Benjamin Callahan, North Carolina State University, Raleigh, NC, USA) for quality filtering, denoising, chimera removal, and feature table construction. Quality filtering was performed by removing sequences with a quality score below Q20 and those shorter than 200 bp. Chimeric sequences were detected and removed using the UCHIME algorithm. Adapter sequences were trimmed using Cutadapt (version 3.4), and non-bacterial sequences were identified and excluded based on alignment with the SILVA database (version 138). Singletons (OTUs appearing only once across all samples) were filtered out to minimize sequencing artifacts.

### 2.6. Statistical Analysis

Community diversity parameters (Shannon, Sobs, Ace, and Chao1 indices) were used to conduct alpha diversity analyses using the mothur software (version 1.44.3) [49]. Sequencing data quality was evaluated by assessing total read counts, read length consistency, and base quality scores. After quality filtering and chimera removal, all samples retained more than 30,000 high-quality reads, with an average Phred score exceeding Q30, indicating reliable base-calling accuracy and sequencing depth for diversity analyses. These parameters ensured the robustness of alpha diversity estimates across all land-use types, and no significant variation in sequencing depth was observed among the samples. All diversity indices were calculated using unrarefied OTU tables clustered at 97% sequence similarity. Beta diversity was analyzed using R software (version 4.2.2; R Core Team, Vienna, Austria), and microbial community differences among land-use types were assessed via one-way analysis of variance (ANOVA) with appropriate post-hoc comparisons. Statistical analyses were conducted using Microsoft Excel 2007 (Redmond, WA, USA) and SPSS 22.0 (IBM, Inc., Armonk, NY, USA). Microbial functions were predicted based on 16S rRNA gene taxonomic assignments using FAPROTAX (version 1.2, updated March 2018) and FUNGuild (version 1.1). The FAPROTAX annotation was performed using the default script provided by the developers, based on OTUs clustered at 97% sequence similarity. For FUNGuild, functional assignments were filtered to retain only entries with confidence rankings of “Probable” and “Highly Probable” to ensure reliability of the predictions. BugBase (https://bugbase.cs.umn.edu/, accessed on 23 May 2024) was employed to infer bacterial phenotypes using OTU tables clustered at 97% similarity [50]. All functional predictions were based on curated databases provided by the respective tools. Output tables were standardized by predicted 16S copy number, and microbial phenotypes were categorized into groups such as Aerobic, Anaerobic, Stress-tolerant, Gram-negative, Gram-positive, Contains Mobile Elements, Facultatively Anaerobic, and Potentially Pathogenic [51,52].

## 3. Results

### 3.1. Soil Physico-Chemical Properties

Soil physicochemical properties exhibited significant variations among different land-use types (Table 1). Soil pH values ranged from 6.33 (SRM) to 6.72 (NF), indicating weak acidity overall, with no significant differences detected among land-use types. The SWC significantly differed across land-use types (*p* < 0.05), with SRM and MRM showing significantly higher water contents (48.01% and 47.57%, respectively) compared to SF, MF, NF, and PF. SOC varied significantly among land-use types (*p* < 0.05), ranging from 19.75 mg·kg^−1^ in MF to 39.39 mg·kg^−1^ in MRM. The SOC levels were notably higher in MRM, NF, and SF, whereas MF exhibited significantly lower SOC levels. The TN contents also significantly varied among land-use types (*p* < 0.05), with the highest concentration observed in MRM (4.88 g·kg^−1^) and the lowest in MF (1.85 g·kg^−1^). DOC contents were highest in NF and PF soils, with values of 367.30 and 353.71 mg·kg^−1^, respectively, significantly greater than those in SF, MF, SRM, and MRM. MBC and MBN also differed significantly among land-use types (*p* < 0.05). The NF and PF soils exhibited significantly higher MBC and MBN contents compared to agricultural soils, with MF showing the lowest MBC and MBN values. NH_4_^+^-N and NO_3_^−^-N concentrations were significantly higher in NF soils compared to other land-use types (*p* < 0.05), reaching 77.01 mg·kg^−1^ and 24.79 mg·kg^−1^, respectively. Agricultural soils exhibited lower nitrogen contents, particularly SF, with NH_4_^+^-N and NO_3_^−^-N concentrations of only 9.71 mg·kg^−1^ and 5.51 mg·kg^−1^, respectively. Soil enzyme activities, including urease, β-glucosidase, and cellulase, also showed significant variations among land-use types (*p* < 0.05). NF soils exhibited the highest urease activity (11,840.17 IU·g^−1^), significantly higher than other land-use types. β-Glucosidase activity was highest in SF (40,336.83 IU·g^−1^) and MRM (39,304 IU·g^−1^), significantly exceeding other land-use types. Cellulase activity was highest in NF soils (230.08 IU·g^−1^), while MF showed the lowest activity (68 IU·g^−1^).

### 3.2. Microbial Alpha and Beta Diversity

Diversity metrics were calculated based on high-quality sequencing data with sufficient read counts across all samples to ensure robust and comparable alpha diversity estimation. Soil bacterial alpha diversity indices exhibited significant variations among different land-use types (Table 2). Specifically, the Sobs index ranged from 1086.32 (PF) to 1721.89 (MRM), with the MRM showing the highest bacterial richness, significantly greater than all other land-use types (*p* < 0.05). In contrast, the PF, NF, and SF soils exhibited the lowest bacterial richness, with no significant differences among these three land use types (*p* > 0.05). Shannon diversity index values varied significantly across land-use types, ranging from 5.26 in NF to 6.43 in MRM. MRM and MF soils had significantly higher Shannon values compared to NF, PF, SF, and SRM. No significant differences were observed among NF, PF, SF, and SRM soils (*p* > 0.05). Ace and Chao1 richness estimators further confirmed these differences. Bacterial richness, as indicated by the ACE index, was significantly higher in MF and MRM compared to other land-use types (*p* < 0.05), suggesting greater microbial community complexity under these conditions. Similarly, Chao1 values were highest in MRM and MF, significantly greater than values from other sites. The PF, NF, SF, and SRM exhibited relatively low Chao1 values, with no significant differences among them.

**Figure 1:**
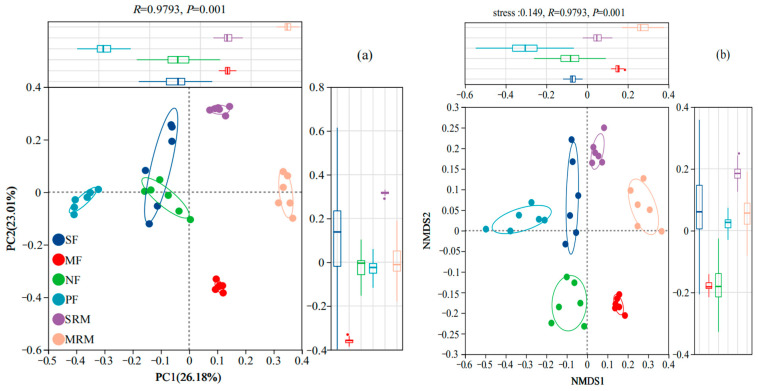
The Principal Coordinate Analysis (**a**) and NMDS (**b**) analysis of soil bacteria at OTU level. Abbreviations, SF, soybean farmland; MF, maize farmland; NF, natural forest; PF, plantation forest; SRM, soybean-retired meadow wetland; MRM, maize-retired marsh wetland. Sample size: *n* = 6 for each land-use type.

The beta diversity of soil bacterial communities across different land-use types was assessed using Principal Coordinate Analysis (PCoA) and Non-Metric Multidimensional Scaling (NMDS) based on OTU composition (Figure 1). Both PCoA (Figure 1a) and NMDS (Figure 1b) plots clearly separated bacterial communities according to land-use types, indicating distinct bacterial assemblages associated with specific land-use practices and ecological conditions. In the PCoA analysis (Figure 1a), the first two principal coordinates explained 36.84% and 19.35% of the variation, respectively, accounting for 56.19% of the total community variation. Bacterial communities from MRM and MF formed distinct clusters on the ordination plot, confirming their compositional divergence from other sites. In contrast, PF, NF, and SF samples grouped more closely, indicating relatively similar microbial communities under forested and agricultural land-use types. Consistently, the NMDS analysis (Figure 1b) further supported these findings, clearly grouping bacterial communities by land-use type and highlighting significant differences in community composition among MRM, MF, and other land use categories. Wetland restoration sites (MRM, SRM) were notably distinct from actively cultivated agricultural soils (SF, MF) and forest ecosystems (NF, PF), reflecting substantial ecological divergence associated with wetland restoration and land-use management practices.

### 3.3. Species Venn Diagram and Soil Bacterial Structure Analysis

**Figure 2:**
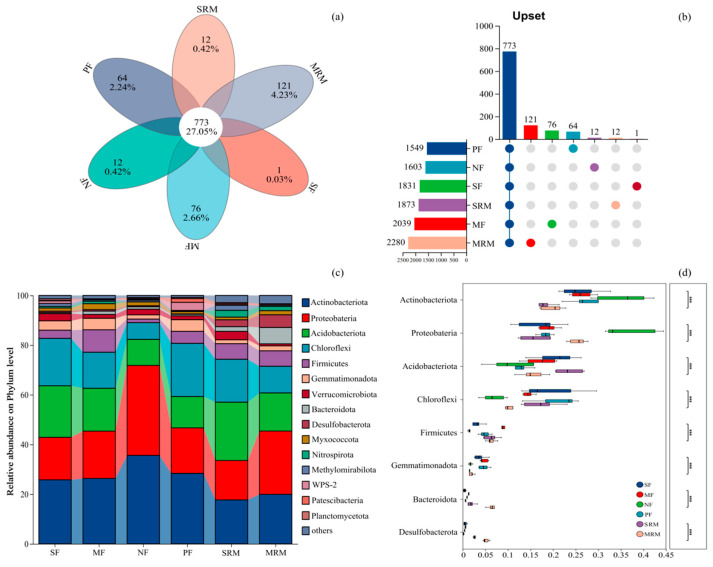
Relative abundance of soil bacterial phyla across land-use types. Abbreviations: SF, soybean farmland; MF, maize farmland; NF, natural forest; PF, plantation forest; SRM, soybean-retired meadow wetland; MRM, maize-retired marsh wetland. (**a**): Venn diagram of soil microorganisms based on the level of OTUs; (**b**): Upset map; (**c**): Relative abundance of soil bacterial communities under different land uses at the phylum level. (**d**): Statistical comparison of the top eight most abundant bacterial taxa. Significance levels: *** *p* < 0.001. Sample size: *n* = 6 for each land-use type.

Soil bacterial community composition exhibited distinct differences across land-use types at both the OTU and phylum levels (Figure 2). The Venn diagram (Figure 2a,b) revealed that a total of 773 operational taxonomic units (OTUs) were shared among all six land-use types, indicating the presence of a relatively stable core microbiota in this cold-region wetland ecosystem. Notably, these shared OTUs accounted for over 75% of the total sequence reads across all samples, suggesting that the core microbiota is dominated by abundant and widely distributed taxa. In contrast, the number of unique OTUs varied considerably among land-use types. MRM exhibited the highest number of unique OTUs (121), followed by MF (76), PF (64), SRM and NF (12 each), and SF (1). The phylum level composition of bacterial communities (Figure 2c,d) demonstrated that *Proteobacteria*, *Actinobacteriota*, *Acidobacteriota*, *Chloroflexi*, and *Firmicutes* were the dominant taxa across all land-use types, but their relative abundances varied significantly. *Proteobacteria* was consistently the most abundant phylum, with notably higher relative abundance in NF and MRM. *Actinobacteriota* exhibited the highest relative abundance in NF and showed a decreasing trend under SRM and MRM. *Chloroflexi* exhibited the highest relative abundance in SF, MF, PF, and SRM soils, but showed a significant decline in NF and MRM. *Bacteroidota* was enriched abundant in MRM.

### 3.4. Correlation Analysis of Environmental Factors

**Figure 3:**
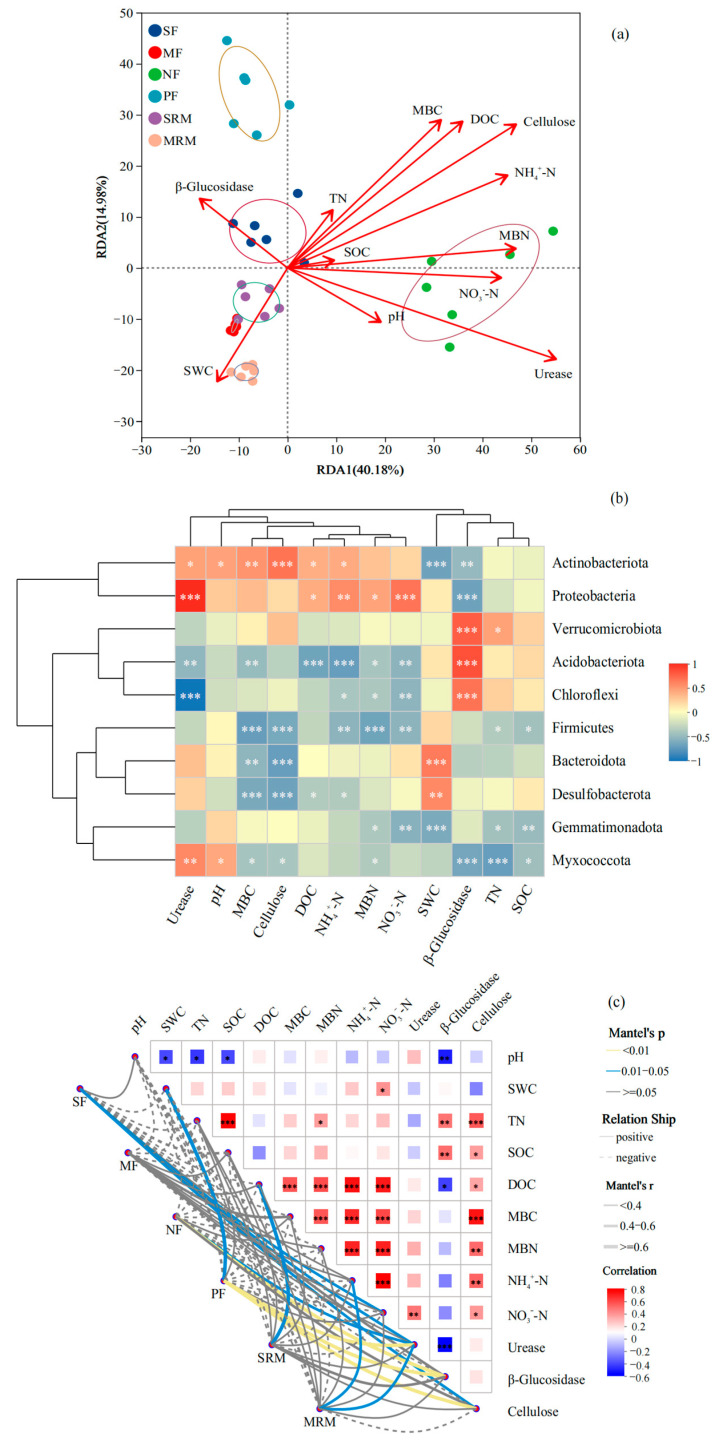
Relationships between microbial community composition and soil properties based on RDA (**a**), heatmap (**b**), and Mantel test (**c**). Mantel test significance was evaluated at α = 0.05. Asterisks indicate significance levels: * *p* < 0.05; ** *p* < 0.01; *** *p* < 0.001. Abbreviations, SF, soybean farmland; MF, maize farmland; NF, natural forest; PF, plantation forest; SRM, soybean-retired meadow wetland; MRM, maize-retired marsh wetland; pH, soil pH value; SOC, soil organic carbon; SWC, soil water content; TN, total nitrogen; DOC, dissolved organic carbon; MBC, microbial biomass carbon; MBN, microbial biomass nitrogen; NH_4_^+^-N, ammonium nitrogen; NO_3_^−^-N, nitrate nitrogen. Sample size: *n* = 6 for each land-use type.

The RDA showed that soil physicochemical properties (Figure 3a), particularly SOC, SWC, pH, and TN, were the main factors associated with shifts in bacterial community composition. The first two RDA axes explained 40.18% and 14.98% of the total variation, respectively. Samples from MRM and SRM were distinctly separated along the positive direction of RDA1 and strongly associated with higher SOC and SWC levels, indicating the important influence of SOC and SWC in restored wetlands. In contrast, SF and MF samples clustered on the negative side of RDA1 and were mainly associated with higher NO_3_^−^-N concentrations and lower SOC levels, reflecting the impact of intensive agricultural practices. NF and PF samples were separated primarily along RDA2 and correlated with higher levels of pH, TN, MBN, and NH_4_^+^-N, suggesting that nitrogen availability and soil acidity play key roles in shaping bacterial communities in forest soils.

The Spearman correlation heatmap (Figure 3b) revealed distinct relationships between dominant bacterial phyla and key environmental factors. In parallel, the Mantel test (Figure 3c), based on a Bray–Curtis dissimilarity matrix for microbial community composition and Euclidean distance for standardized environmental variables, showed significant correlations between bacterial community structure and multiple soil physicochemical and biochemical parameters. *Actinobacteriota* exhibited significant positive correlations with cellulose (*p* < 0.001), MBC (*p* < 0.01), urease, pH, DOC, and NH_4_^+^-N (*p* < 0.05), while showing significant negative correlations with SWC (*p* < 0.001) and β-glucosidase (*p* < 0.01). *Proteobacteria* was positively correlated with urease and NO_3_^−^-N (*p* < 0.001), NH_4_^+^-N (*p* < 0.01), as well as DOC and MBN (*p* < 0.05), but exhibited a significant negative correlation with β-glucosidase (*p* < 0.001). *Verrucomicrobiota*, *Acidobacteriota*, and *Chloroflexi* were all significantly positively correlated with β-glucosidase (*p* < 0.001). In addition, SWC showed significant positive correlations with *Bacteroidota* (*p* < 0.001) and *Desulfobacterota* (*p* < 0.01). *Acidobacteriota* was negatively correlated with DOC and NH_4_^+^-N (*p* < 0.001), and with urease, MBC, and NO_3_^−^-N (*p* < 0.01). *Chloroflexi* exhibited significant negative correlations with urease (*p* < 0.001), NO_3_^−^-N (*p* < 0.01), and NH_4_^+^-N and MBN (*p* < 0.05). Firmicutes showed strong negative correlations with MBC, cellulose, and MBN (*p* < 0.001), and with NO_3_^−^-N and NH_4_^+^-N (*p* < 0.01). Among these, SOC, DOC, SWC, TN, MBC, MBN, NH_4_^+^–N, and pH showed highly significant positive correlations with community structure (*p* < 0.01), with Mantel’s r values generally above 0.6, indicating strong associations (Figure 3c). SOC and DOC exhibited the strongest correlations, suggesting that soil carbon availability plays a central role in shaping microbial community composition across land-use types. Enzyme activities, including urease, cellulase, and β-glucosidase, were also significantly associated with bacterial communities (*p* < 0.05), though the correlation strength was moderate. Notably, NO_3_^−^-N displayed relatively weaker correlations compared to other nitrogen-related variables.

### 3.5. Phenotypic Prediction Analysis

**Figure 4:**
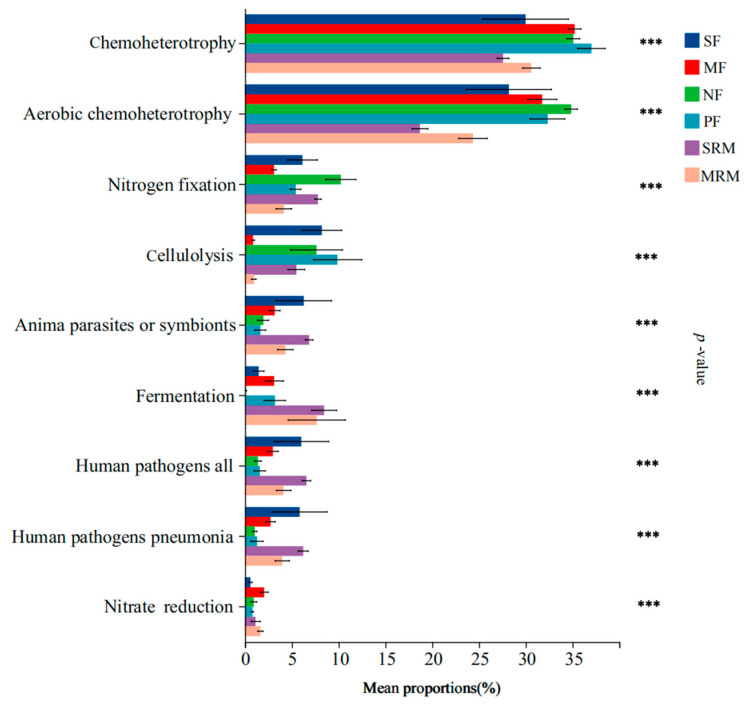
FAPROTAX-based functional prediction across land-use types. Note: significance levels: *** *p* < 0.001. Abbreviations, SF, soybean farmland; MF, maize farmland; NF, natural forest; PF, plantation forest; SRM, soybean-retired meadow wetland; MRM, maize-retired marsh wetland. Sample size: *n* = 6 for each land-use type.

Land-use changes and wetland restoration processes substantially altered the functional composition of soil bacterial communities, particularly affecting pathways related to carbon metabolism, nitrogen cycling, and potential pathogenicity (Figure 4). Functional annotations were filtered to remove low-confidence predictions and rare functions (relative abundance < 0.1%) prior to analysis to ensure reliability. Functions related to chemoheterotrophy and aerobic chemoheterotrophy exhibited the highest relative abundances in MF, NF, and PF soils, significantly exceeding those in SRM and MRM, while no significant differences were found compared to SF. Nitrogen fixation functions were significantly more abundant in SF, NF, and SRM than in other land-use types. Cellulolysis functions showed significantly higher relative abundances in SF, NF, PF, and SRM compared to MF and MRM. Functions associated with animal parasites or symbionts, human pathogens (all types), and human pathogens (pneumonia) were significantly enriched in SF, SRM, and MRM. Fermentation-related functions were most abundant in SRM and MRM, significantly higher than in other land-use types, whereas nitrate reduction functions showed peak abundance in MF.

### 3.6. BugBase Phenotype Prediction

**Figure 5:**
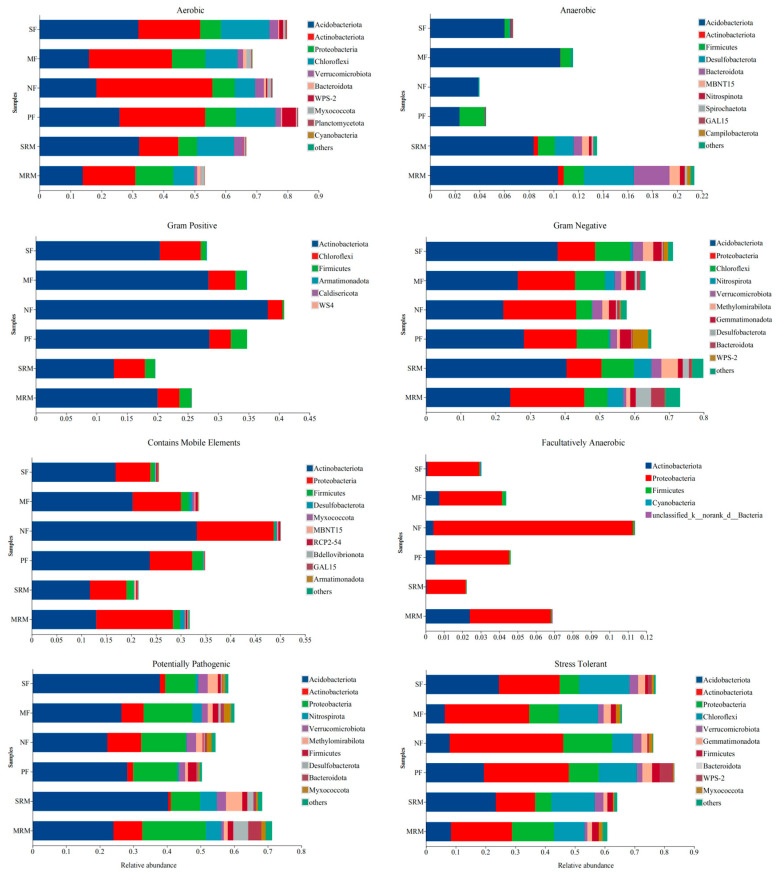
BugBase predicted bacterial phenotypes under different land-use types. Abbreviations, SF, soybean farmland; MF, maize farmland; NF, natural forest; PF, plantation forest; SRM, soybean-retired meadow wetland; MRM, maize-retired marsh wetland. Sample size: *n* = 6 for each land-use type.

The phenotypic abundance analysis of eight types of bacteria (aerobic, anaerobic, Gram-positive, Gram-negative, contains mobile element, facultatively anaerobic, potentially pathogenic, and stress-tolerant) predicted by BugBase based on different land use patterns is presented in Figure 5. Soil aerobic metabolism functions were mainly dominated by *Acidobacteriota* and *Actinobacteriota*, with notable differences among land-use types. *Actinobacteriota* contributed most to aerobic functions in SF, MF, NF, and PF, but was relatively lower in SRM and MRM. Anaerobic metabolism-associated bacteria showed the highest relative abundances in SRM and MRM, mainly contributed by *Acidobacteriota*, *Firmicutes*, *Desulfobacterota*, *Bacteroidota,* and *Actinobacteriota*. Compared to wetlands and agricultural soils, forest soils (NF, PF) exhibited lower anaerobic metabolic potential. For Gram-positive phenotypes, *Actinobacteriota* contributed most in SF, MF, SRM, and MRM, but less in NF and PF. Gram-negative bacteria dominated across all sites, with the highest relative abundances observed in SF and SRM, primarily driven by *Proteobacteria* and *Acidobacteriota*. *Proteobacteria* and *Actinobacteriota* were the major contributors to the bacterial communities that contain mobile elements. Among the different land-use types, NF exhibited the highest relative abundance of bacteria harboring mobile genetic elements, whereas SRM showed the lowest. Facultatively anaerobic bacteria were primarily contributed by *Proteobacteria*. Among the different sites, NF had the highest relative abundance, while SRM showed the lowest. The potentially pathogenic bacterial communities were mainly contributed by *Acidobacteriota* and *Proteobacteria*. Wetland restoration soils (SRM, MRM) exhibited higher relative abundances of potentially pathogenic bacteria compared to agricultural (SF, MF) and forest soils (NF, PF). Among all sites, SRM showed the highest abundance of potentially pathogenic taxa. The data show that *Proteobacteria*, *Actinobacteriota*, and Chloroflexi were the dominant contributors to the stress-tolerant phenotype, with slightly higher proportions in forest soils (FW, AFL) than in agricultural (SS, RS) and wetland restoration soils (MR, MS).

## 4. Discussion

### 4.1. Relationship Between Soil Physicochemical Properties and Diversity

This study demonstrates that variation in bacterial alpha diversity across different land-use types in cold-region wetlands is not solely driven by a few primary soil parameters (e.g., SWC, SOC, TN, pH), but also significantly shaped by a broader suite of biochemical indicators such as DOC, MBC, MBN, NH_4_^+^-N, NO_3_^−^-N, and key enzyme activities. These findings align with the broader understanding in microbial ecology that diversity is modulated by both resource quantity and quality [53,54]. For instance, elevated DOC in restored and natural wetlands supports greater microbial niche differentiation by enhancing substrate heterogeneity—providing a broader range of carbon compounds that can be selectively utilized by different microbial taxa with distinct metabolic capacities. This aligns with ecological theories linking carbon availability to niche partitioning and community complexity [55]. Furthermore, microbial biomass indicators (MBC and MBN) reflect not only microbial abundance but also ecosystem nutrient cycling potential, and their association with diversity in this study supports the notion that microbial community structure is closely tied to ecosystem function [56,57]. This is particularly relevant in cold-region wetlands, where biomass accumulation is often constrained by short growing seasons and fluctuating redox conditions [58]. The influence of NH_4_^+^-N and NO_3_^−^-N suggests that shifts in nitrogen form availability under different land uses may favor different functional guilds, in line with recent studies emphasizing nitrogen form as a selective force in microbial assembly [59]. Moderate nitrogen levels supported microbial diversification, while nitrogen-deficient conditions favored narrow, specialized communities [60]. In contrast, high nitrogen availability in fertilized fields was associated with reduced evenness, potentially due to the overrepresentation of nitrophilic taxa such as Nitrosomonas, Pseudomonas, and other fast-growing members of the *Proteobacteria* phylum that thrive under nitrogen-enriched conditions [61]. Enzyme activities serve as functional proxies for microbial capacity in biogeochemical cycling [62]. Urease activity, linked to nitrogen mineralization, was enhanced in restored wetlands, indicating a shift toward active nitrogen turnover. β-glucosidase, associated with cellulose degradation, was positively related to microbial richness, likely due to its role in processing plant-derived carbon. Similarly, cellulase activity was pronounced in organic-rich soils, reinforcing the connection between substrate complexity and microbial functional diversity [63].

These findings suggest that soil biochemical attributes, in tandem with physical and chemical properties, jointly determine bacterial alpha diversity. Restored wetland soils, which offer balanced hydrological conditions, sufficient carbon input, and moderate nutrient availability, support more complex and functionally diverse microbial assemblages [64]. Conversely, forested and intensively managed agricultural soils, characterized by nutrient limitation or community homogenization, tend to host less diverse microbial communities [65]. Integrating microbial, chemical, and enzymatic indicators in ecological assessments provides a more comprehensive understanding of the soil microbiome’s response to land-use change [66]. These insights are essential for optimizing wetland restoration strategies that aim to recover not only vegetation and hydrology but also the microbial ecological foundation of soil systems [67].

### 4.2. Environmental Factors Influencing Soil Microbial Community Structure

The distinct variations observed in soil bacterial community composition across different land-use types reflect the critical regulatory role of soil physicochemical and biochemical properties in structuring microbial assemblages in cold-region wetlands [68]. The consistent presence of a core microbiota among all land-use types suggests that a fundamental ecological baseline persists, despite varying environmental conditions, supporting overall system resilience [69]. The higher number of unique OTUs identified in MRM indicates intensified niche differentiation and microbial specialization, likely driven by enhanced environmental heterogeneity following wetland restoration. Restoration processes increase organic matter accumulation, moisture stability, and redox variability, creating microsites that promote the coexistence of diverse microbial taxa with distinct functional attributes [70]. This suggests that wetland restoration enhances not only microbial community structure but also functional redundancy, both of which were statistically significant across restored versus non-restored land-use types (*p* < 0.05 for alpha diversity and several functional traits). These consistent shifts imply that restoration contributes to more diverse and functionally buffered microbial communities. Although ecosystem multifunctionality was not directly measured, the observed increase in predicted functional trait diversity suggests greater potential for sustaining multiple biogeochemical processes [71]. At the phylum level, variations in the relative abundances of *Proteobacteria*, *Actinobacteriota*, *Acidobacteriota*, *Chloroflexi*, and *Firmicutes* reveal divergent microbial strategies under different soil environments. The dominance of *Proteobacteria* in restored and agricultural soils reflects their metabolic plasticity and preference for nutrient-rich and moisture-variable conditions [72]. In contrast, the enrichment of *Actinobacteriota* in forest soils suggests adaptation to oligotrophic environments with relatively higher cellulose content and ammonium nitrogen availability, highlighting resource-driven microbial selection processes [73].

Redundancy analysis identified SOC, SWC, pH, and TN as principal drivers of bacterial community differentiation. Higher SOC and SWC levels in restored wetlands correlated with distinct microbial compositions, indicating that increased carbon substrates and moisture availability promote more complex and stratified microbial networks [74]. In agricultural soils, elevated NO_3_^−^-N concentrations, coupled with lower SOC, were selected for fast-growing copiotrophic taxa, reducing community evenness and increasing the risk of functional homogenization [75]. Environmental correlations further highlighted the role of microbial functional activities in shaping community structures [76]. Enzyme activities, particularly β-glucosidase, urease, and cellulase, emerged as important indicators linking substrate turnover processes to microbial community assembly [66]. High β-glucosidase activity associated with *Acidobacteriota* and *Chloroflexi* suggests enhanced carbon degradation pathways in restored and agricultural soils, contributing to dynamic carbon cycling and influencing microbial niche differentiation [77]. Mantel test results confirmed that bacterial community patterns are strongly coupled to soil carbon pools (SOC, DOC), nitrogen status (TN, NH_4_^+^-N, NO_3_^−^-N), and SWC. These findings highlight that soil microbial community structure in cold-region wetlands is co-regulated by resource quantity (carbon and nitrogen) and resource quality (substrate availability and environmental redox potential). Restoration strategies that optimize these parameters are therefore likely to contribute to the reestablishment of diverse microbial communities with improved ecological capacity. Although microbial resilience was not directly measured, the maintenance of core taxa across land-use types, combined with significantly higher Shannon diversity and richness indices (e.g., Chao1 and ACE) observed in restored soils, suggest improved adaptability and functional stability under fluctuating environmental conditions [78,79]. These results collectively demonstrate that wetland restoration alters soil physicochemical gradients, which in turn promote microbial community diversification—reflected not only in OTU counts but also in elevated richness and evenness metrics—and facilitate functional reorganization [80]. Understanding the mechanistic pathways by which environmental factors shape microbial assembly provides essential insights for guiding effective wetland conservation and management in cold region ecosystems [81].

### 4.3. Functional Prediction Analysis of Soil Bacterial Communities

The predicted functional composition of soil bacterial communities showed substantial variation across land-use types, underscoring the influence of environmental gradients on microbial metabolic strategies [82]. Land-use conversion and wetland restoration notably affected functions related to carbon metabolism, nitrogen cycling, and potential pathogenicity, corresponding with shifts in microbial community structure [83]. Chemoheterotrophy and aerobic chemoheterotrophy dominated in MF, NF, and PF soils, reflecting conditions that favor oxygen-dependent carbon degradation [84]. In contrast, fermentation-related functions were enriched in wetland restoration soils (SRM and MRM), consistent with anaerobic, saturated conditions promoting reductive metabolism [85]. Nitrogen fixation functions were relatively more abundant in SF, NF, and SRM soils, likely due to hydrological restoration and legume-associated microbial communities enhancing nitrogen inputs [86,87]. Elevated nitrate reduction functions in MF soils aligned with increased NO_3_^−^-N levels, indicating a shift toward denitrification pathways under fertilized conditions [88]. Predicted functions associated with potentially pathogenic bacteria were more abundant in SRM and MRM compared to forest and agricultural soils and were primarily attributed to members of *Acidobacteriota* and *Proteobacteria*. However, these interpretations should be approached cautiously. The annotations were based on phenotype-inference tools (e.g., BugBase), which rely on taxonomic profiles rather than direct evidence of virulence gene presence or activity. Thus, the observed increase may reflect shifts in community ecological strategies under transitional conditions rather than an actual elevation in pathogenic risk. Further validation using metagenomic sequencing or targeted functional gene assays is required. Nonetheless, this finding underscores the importance of microbial monitoring during restoration to anticipate potential unintended consequences for soil or plant health [89].

Phenotypic predictions via BugBase revealed additional ecological patterns. Aerobic phenotypes dominated agricultural and forest soils, whereas anaerobic traits were enriched in restored wetlands, supporting previous findings of fermentative metabolism under low redox conditions [83,90]. Gram-negative bacteria—largely affiliated with *Proteobacteria*—were prevalent across all soils, with relatively higher proportions in agricultural and restored sites, suggesting microbial assemblages adapted for membrane flexibility under nutrient and moisture fluctuations [91]. Bacteria harboring mobile genetic elements were most abundant in natural forest soils, potentially reflecting higher rates of horizontal gene transfer in less disturbed environments [92]. Facultative anaerobes and stress-tolerant phenotypes were more common in forest soils, likely due to oligotrophic but relatively stable environmental conditions selecting for metabolically flexible taxa [93]. Overall, functional predictions indicate that soil microbial communities in cold-region wetlands are highly responsive to environmental factors such as moisture, carbon input, and nutrient dynamics. Restoration enhances not only taxonomic diversity but also metabolic versatility, contributing to potential ecosystem multifunctionality under fluctuating conditions [94]. While FAPROTAX and BugBase provide useful insights based on 16S rRNA gene data, these predictions reflect potential, not actual function. Limitations include the inability to detect gene expression, functional redundancy, or strain-level variation. Future research will incorporate targeted qPCR of key functional genes and shotgun metagenomic sequencing to validate functional capacity and improve mechanistic understanding of microbial roles in wetland recovery.

## 5. Conclusions

This study highlights the critical role of soil properties, particularly moisture, organic carbon, nitrogen, and enzyme activity, in regulating microbial diversity and function across land-use types in cold-region wetlands. Restoration improved microbial diversity and functional redundancy by enhancing soil organic matter and water content, promoting carbon and nitrogen cycling and microbial adaptability. Functional predictions revealed a shift from aerobic to anaerobic and fermentative pathways in restored sites, with a temporary rise in opportunistic pathogenic potential. These findings underscore the importance of managing soil conditions during restoration to support beneficial microbial functions. To enhance ecosystem recovery, restoration should maintain adequate soil moisture and organic inputs through rewetting and reduced disturbance. Microbial indicators such as β-glucosidase activity and nitrogen cycling functions can serve as early signals of restoration progress. As functional profiles were inferred from 16S rRNA data, further validation using metagenomics and qPCR is recommended. Overall, the results provide actionable guidance for microbial-informed restoration practices aimed at improving soil ecological resilience in cold-temperate wetlands.

## Figures and Tables

**Table 1:** Physical and chemical properties of soil in land-use type.

Variables	SF	MF	NF	PF	SRM	MRM
pH	6.43 ± 0.07 a	6.71 ± 0.11 a	6.72 ± 0.33 a	6.41 ± 0.11 a	6.33 ± 0.30 a	6.42 ± 0.08 a
SWC (%)	26.75 ± 2.11 b	26.55 ± 4.25 b	30.07 ± 1.69 ab	32.79 ± 1.81 ab	48.01 ± 2.95 a	47.57 ± 2.77 a
SOC (mg·kg^−1^)	32.07 ± 1.75 a	19.75 ± 1.77 b	30.12 ± 5.41 a	25.52 ± 6.40 ab	25.32 ± 7.80 ab	39.39 ± 6.64 a
TN (g·kg^−1^)	3.46 ± 0.46 a	1.85 ± 0.84 b	3.54 ± 0.19 a	3.31 ± 0.34 a	2.54 ± 0.85 a	4.88 ± 1.42 a
DOC (mg·kg^−1^)	204.98 ± 8.23 c	233.95 ± 8.44 b	367.30 ± 25.41 a	353.71 ± 17.47 a	247.64 ± 11.49 b	234.72 ± 10.19 b
MBC (mg·kg^−1^)	1437.38 ± 149.91 b	866.19 ± 136.53 c	1723.13 ± 138.01 a	1655.94 ± 164.87 a	1333.40 ± 122.41 b	1380.75 ± 112.92 b
MBN (mg·kg^−1^)	93.68 ± 6.56 c	66.93 ± 5.31 d	230.08 ± 26.51 a	130.35 ± 16.37 b	122.41 ± 20.54 b	129.24 ± 22.41 b
NH_4_^+^-N (mg·kg^−1^)	9.71 ± 1.34 d	11.26 ± 3.09 d	77.01 ± 5.11 a	43.92 ± 2.80 b	24.92 ± 1.47 c	24.08 ± 1.47 c
NO_3_^−^-N (mg·kg^−1^)	5.51 ± 0.79 c	3.77 ± 0.81 c	24.79 ± 3.75 a	13.68 ± 2.77 b	14.46 ± 2.23 b	11.99 ± 1.50 b
Urease (IU·g^−1^)	6333.17 ± 164.73 c	6578.33 ± 368.80 c	11,840.17 ± 879.52 a	5583 ± 652.96 c	7663.67 ± 498.84 b	5881.83 ± 499.07 c
β-Glucosidase (IU·g^−1^)	40,336.83 ± 985.85 a	25,559 ± 1035.71 c	26,542.50 ± 1591.47 c	30,732.83 ± 794.36 b	26,515 ± 1598.66 c	39,304 ± 1320.78 a
Cellulose (IU·g^−1^)	158.33 ± 21.85 b	68 ± 6.68 c	230.08 ± 26.51 a	130.35 ± 16.37 b	122.41 ± 20.54 b	129.24 ± 22.41 b

Note: values represented mean ± standard deviations (*n* = 6). Different letters stand for significant effects (*p* < 0.05). Abbreviations: pH, soil pH value; SOC, soil organic carbon; SWC, soil water content; TN, total nitrogen; DOC, dissolved organic carbon; MBC, microbial biomass carbon; MBN, microbial biomass nitrogen; NH_4_^+^-N, ammonium nitrogen; NO_3_^−^-N, nitrate nitrogen; SF, soybean farmland; MF, maize farmland; NF, natural forest; PF, plantation forest; SRM, soybean-retired meadow wetland; MRM, maize-retired marsh wetland.

**Table 2:** The alpha diversity of soil bacteria in land-use type.

Soil Sample	Sobs	Shannon	Ace	Chao 1
SF	1181.35 ± 109.18 d	5.69 ± 0.18 b	1364.93 ± 128.39 c	1381.93 ± 135.99 b
MF	1590.53 ± 50.78 b	6.32 ± 0.07 a	1765.72 ± 40.44 a	1812.32 ± 35.18 a
NF	1156.34 ± 91.26 d	5.26 ± 0.29 b	1334.45 ± 70.51 c	1348.37± 77.21 b
PF	1086.32 ± 141.33 d	5.65 ± 0.21 b	1228.32 ± 165.85 c	1256.52 ± 161.71 b
SRM	1365.82 ± 73.49 c	5.88 ± 0.05 b	1559.94 ± 88.59 b	1595.66 ± 105.79 b
MRM	1721.89 ± 56.71 a	6.43 ± 0.11 a	1765.77 ± 40.44 a	1956.14 ± 105.79 a

Note: values represented mean ± standard deviations (*n* = 6). Different letters stand for significant effects (*p* < 0.05). Abbreviations: SF, soybean farmland; MF, maize farmland; NF, natural forest; PF, plantation forest; SRM, soybean-retired meadow wetland; MRM, maize-retired marsh wetland.

## Data Availability

The datasets generated for this study can be accessed from the NCBI Sequence Read Archive under accession number PRJNA1271745.
